# Breaking the borders of wakefulness and sleep—the search for novel biomarkers to quantify sleepiness

**DOI:** 10.1093/sleep/zsae167

**Published:** 2024-07-24

**Authors:** David R Schreier, Veronique E C Vael, Gert Jan Lammers

**Affiliations:** Department of Anesthesia, Critical Care and Pain Medicine, Massachusetts General Hospital and Harvard Medical School, Boston, MA, USA; Picower Institute for Learning and Memory, Massachusetts Institute of Technology, Cambridge, MA, USA; Center for Neurotechnology and Neurorecovery, Department of Neurology, Massachusetts General Hospital and Harvard Medical School, Boston, MA, USA; Department of Neurology, Inselspital, Bern University Hospital, and University of Bern, Switzerland; Stichting Epilepsie Instellingen Nederland (SEIN), Sleep-Wake Centre, Heemstede, The Netherlands; Stichting Epilepsie Instellingen Nederland (SEIN), Sleep-Wake Centre, Heemstede, The Netherlands; Department of Neurology, Leiden University Medical Centre, Leiden, The Netherlands; Stichting Epilepsie Instellingen Nederland (SEIN), Sleep-Wake Centre, Heemstede, The Netherlands; Department of Neurology, Leiden University Medical Centre, Leiden, The Netherlands

Excessive daytime sleepiness (EDS) affects up to 33% of the population, and can have serious consequences for work performance, social interaction, and driving ability [1–3]. In addition, it significantly increases the economic costs on society [4–6]. In most cases, EDS results from insufficient sleep or disruption of sleep at night (e.g. severe sleep apnea), the main therapeutic focus in these cases is on improving nighttime sleep. In central disorders of hypersomnolence, the etiology can be traced less unambiguously to disturbed nighttime sleep. Despite the high impact of EDS, we still do not have reliable biomarkers to distinguish EDS caused by insufficient sleep from EDS caused by central disorders of hypersomnolence. To determine the capacity for wakefulness and for treatment monitoring, sleep onset latency (SOL) in the maintenance of wakefulness test (MWT) is widely used [7]. However, motivation can affect the SOL and limit predictions of EDS-related real-world risks [7, 8]. Moreover, relevant incidents occur primarily during periods requiring both wakefulness and attention [9]. Therefore, we should not exclusively focus on the quantification of sleepiness and also assess sustained attention.

Sleepiness, in simple terms, is the biological drive to sleep [10]. In reality, sleepiness embodies a multifaceted, complex, and theoretical construct that is based on sleep need and the capacity to resist that need and maintain wakefulness. Although sleep–wake recordings are not on paper anymore, the official scoring criteria are still limited to epochs of 30 seconds [11]. In addition to this temporal limitation, the electroencephalogram (EEG) scoring is spatially restricted to one global state of the brain between wakefulness and any sleep stage and therefore binary; one is either awake or asleep. This is in stark contrast to evidence demonstrating that both sleep and wakefulness can be local and therefore coexist [12, 13]. Consequently, present clinical definitions of sleep–wake states are at least partially limiting the availability of tools to measure and quantify sleepiness in the borderland between wakefulness and sleep. On the other hand, novel concepts on the definition of sleepiness and technological advances facilitate the development of new quantitative measures for sleepiness [14]. This could be particularly relevant for people with narcolepsy, where transitions between wakefulness and sleep are more fluid, more frequent, and occur both during the day and the night.

In this issue of *SLEEP*, Tracey et al. [15] are exploring whether microsleep episodes (MSEs) and computational approaches to EEG analysis in the MWT could provide biomarkers for sleepiness that go beyond the SOL, and break with the traditional borders of wakefulness and sleep. They have analyzed data from a phase 1B trial in people with NT1 and narcolepsy type 2 (NT2) that were randomized to different doses of TAK-925, a selective orexin receptor 2 agonist, or placebo (NCT03748979). The remarkable improvement of EDS induced by TAK-925, offered the opportunity to study potential sleepiness biomarkers. People with NT1 received lower TAK-925 doses (11 and 44 mg) than people with NT2 (44 and 112 mg), that were infused daily (9 hours) over 7 days. The MWT was performed at baseline, days 1 and 7 of treatment, and MSEs (3–15 seconds) were scored by blinded experts who reviewed EEG (frontal, central, and occipital), EOG, and EMG. 15-second estimates of “hypnodensities” were computed from MWT data using a previously published neural network (trained and validated on polysomnography [PSG] data) [16] to create a “sleepiness score,” and a multitaper spectral analysis [17] of O2-A1 was performed to compute θ/α. They conclude that all three indeed are biomarkers for (increasing) sleepiness.

As expected, SOL was significantly longer for all participants treated with TAK-925 compared to baseline or placebo. For most intermediate SOLs, the third MWT trial was shorter compared to the other trials (“siesta dip”) while this effect rarely occurred for SOL that were very short or set to 40 minutes. The authors’ explanation of an “unmasking effect” due to treatment, assuming a floor effect in untreated narcolepsy, is in line with earlier descriptions of less severe EDS and a ceiling effect in healthy controls [18]. The floor effect might also explain that MSEs were present in less than half of the trials at baseline (NT1: 40%, NT2: 48%). Most likely, the overwhelming sleepiness in NT1 and NT2 more often results in a direct transition to sleep than MSEs prior to sleep onset. Consequently, it can be speculated that MSEs only occur if the capacity to maintain wakefulness (including motivation to stay awake) is sufficient to counteract short periods of sleep. Accordingly, the MSEs should occur more frequently under treatment with a stimulant; unless the treatment is successful enough to introduce a ceiling effect. Although some ceiling effect for SOL is visible in the data of Tracey et al., especially for high-dose treatment in NT1, it remains unclear if this is sufficient to explain the lower number of overall trials with MSEs since extended wakefulness periods after treatment may provide an opportunity to unmask MSEs. This challenges the conclusion of Tracey et al. that the MSE rate appears to be a sensitive treatment marker. Moreover, MSEs < 3 seconds were not included and the MSE rate was calculated based on the number of MSEs per 30-second epoch for the total trial duration despite previous evidence demonstrating that 40% of MSEs are 1–3 seconds long and MSEs most often occur within a few minutes of sleep onset [19]. Therefore, a higher temporal scoring resolution (<3 seconds) might have increased the number of trials with MSEs and thus sensitivity. If MSEs should be considered a “biomarker” they have to be clearly defined. Although more detailed criteria for visual scoring of EEG-based MSEs in the MWT have been proposed [19], the criteria to define MSEs mostly vary across studies and complicate the interpretation of results across studies. Standardization by integration of MSE scoring criteria into official guidelines, for example, the AASM manual for the Scoring of Sleep and Associated Events, could mitigate this problem. Ideally, a MSEs definition would not only provide criteria for the scoring of neurophysiological data (EEG, EOG, and EMG).

Aside from visual or automated detection of MSE, the exploration of continuous variables determining state probabilities and spectral analysis is of interest. Tracey et al. reported that θ/α, limited to one occipital channel, seemed slightly more reliable than the sleepiness score and values for both mostly increased after lights off. Although the reported results seem promising, the authors acknowledge the θ/α to be “noisy” and the sleepiness score to be a “black box” method. It might be important to note that the sleepiness score was developed based on polysomnography data during which, in contrast to the MWT, the eyes are mainly closed and participants are not instructed to maintain wakefulness. To which extent the different conditions might affect the reported findings is unclear but should be explored in future.

In conclusion, the results of Tracey et al. challenge the present criteria of scoring sleep (onset) and wakefulness, and deserve credit for illustrating how future biomarkers, such as MSEs and computed state probabilities, might replace them. However, to eventually break the borders between wakefulness and sleep, it is necessary to replicate results in larger and more heterogeneous groups beyond narcolepsy, include measures of sustained attention, and continue developing low-cost and straightforward technology for implementation.
